# Use of thyroid hormones in hypothyroid and euthyroid patients: a THESIS* survey of Belgian specialists *THESIS: treatment of hypothyroidism in Europe by specialists: an international survey

**DOI:** 10.1186/s13044-022-00121-9

**Published:** 2022-03-05

**Authors:** Maria-Cristina Burlacu, Roberto Attanasio, Laszlo Hegedüs, Endre V. Nagy, Enrico Papini, Petros Perros, Kiswendsida Sawadogo, Marie Bex, Bernard Corvilain, Chantal Daumerie, Brigitte Decallonne, Damien Gruson, Bruno Lapauw, Rodrigo Moreno Reyes, Patrick Petrossians, Kris Poppe, Annick Van den Bruel, David Unuane

**Affiliations:** 1 Department of Endocrinology and Nutrition, Cliniques Universitaires St-Luc, Université Catholique de Louvain, 1200 Brussels, Belgium; 2 IRCCS Orthopedic Institute Galeazzi, Endocrine Unit, 20161 Milan, Italy; 3 Department of Endocrinology, Odense University Hospital, University of Southern Denmark, Odense, Denmark; 4 Division of Endocrinology, Department of Medicine, Faculty of Medicine, University of Debrecen, Debrecen, Hungary; 5 Department of Endocrinology and Metabolism, Regina Apostolorum Hospital, Albano, Rome, Italy; 6 Department of Endocrinology, Royal Victoria Infirmary, Newcastle upon Tyne, UK; 7 Statistical support unit, Institut Roi Albert II, Cliniques universitaires Saint-Luc, Brussels, Belgium; 8 Department of Endocrinology, University Hospitals Leuven, Leuven, Belgium; 9 Department of Endocrinology, Erasme University Hospital, Université Libre de Bruxelles, Brussels, Belgium; 10 Department of Clinical Biochemistry, Cliniques Universitaires St-Luc, Université Catholique de Louvain, Brussels, Belgium; 11 Department of Endocrinology, Ghent University Hospital, Ghent, Belgium; 12 Department of Nuclear Medecine, Erasme University Hospital, Université Libre de Bruxelles, Brussels, Belgium; 13 Department of Endocrinology, CHU de Liège, Université de Liège, Liège, Belgium; 14 Endocrine Unit, CHU Saint- Pierre, Université Libre de Bruxelles (ULB), Brussels, Belgium; 15 Department of Endocrinology, AZ Sint Jan Brugge Oostende AV, Brugge, Belgium; 16 Department of Internal Medicine, Endocrine Unit, UZ Brussel, Vrije Universiteit Brussel (VUB), Brussels, Belgium

**Keywords:** Hypothyroidism, Survey, Thyroid hormones

## Abstract

**Background:**

Hypothyroidism is a topic that continues to provoke debate and controversy with regards to specific indications, type of thyroid hormone substitution and efficacy. We investigated the use of thyroid hormones in clinical practice in Belgium, a country where currently only levothyroxine (LT4) tablet formulations are available.

**Method:**

Members of the Belgian Endocrine Society were invited to respond to an online questionnaire. Results were compared with those from other THESIS surveys.

**Results:**

Eighty (50%) of the invited 160 individuals, completed the questionnaire. LT4 was the first treatment of choice for all respondents. As secondary choice, some also prescribed liothyronine (LT3) and LT4 + LT3 combinations (2 and 7 respondents, respectively). Besides hypothyroidism, 34 and 50% of respondents used thyroid hormones for infertile euthyroid TPOAb positive women and the treatment of a growing non-toxic goiter, respectively. Had alternative formulations of LT4 to tablets been available (soft gel or liquid L-T4), 2 out of 80 (2.5%) participants would consider them for patients achieving biochemical euthyroidism but remaining symptomatic. This proportion was higher in case of unexplained poor biochemical control of hypothyroidism (13.5%) and in patients with celiac disease or malabsorption or interfering drugs (10%). In symptomatic euthyroid patients, 20% of respondents would try combined LT4 + LT3 treatment. Psychosocial factors were highlighted as the main contributors to persistent symptoms.

**Conclusions:**

LT4 tablets is the preferred treatment for hypothyroidism in Belgium. A minority of the respondents would try combined LT4 + LT3 in symptomatic but biochemically euthyroid patients. Thyroid hormones are prescribed for euthyroid infertile women with thyroid autoimmunity and patients with non-toxic goiter, a tendency noted in other European countries, despite current evidence of lack of benefit.

**Supplementary Information:**

The online version contains supplementary material available at 10.1186/s13044-022-00121-9.

## Introduction

**Fig. 1:**
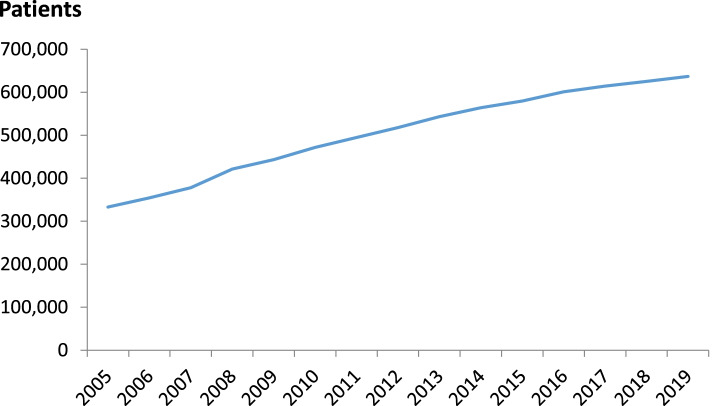
Number of Belgian patients treated by year with thyroid hormones between 2005 and 2019. Source: National Institute of Diseases (INAMI-RIZIV, https://www.inami.fgov.be)

Hypothyroidism is the consequence of insufficient thyroid hormone (TH) action on target tissues [1]. Five percent of the European population, increasing with advancing age, has some degree of hypothyroidism [2]. In Belgium in 2012, more than 500,000 patients were treated with TH [3]. This number has been increasing (Fig. 1), a trend also noted in other European countries [4]. The thresholds of serum TSH when initiating TH treatment are falling, and this may be driven by attributing common and non-specific symptoms such as weight gain, fatigue, mood disorders and poor memory to hypothyroidism [5, 6]. The unjustified prescription of TH could partially explain why a significant proportion of the treated patients are not satisfied with their treatment, but the efficacy and tolerability of different TH formulations or combinations have also been implicated [7, 8].

New LT4 formulations (liquid and soft gel capsules), approved by the European Medicines Agency and the American Food and Drug Administration, are claimed to improve bioavailability, presumably by facilitating absorption, but large-scale randomized studies are needed before these formulations can be prioritized over classical ones, at least in patients without gastrointestinal diseases and/or no concomitant medications [9]. Moreover, these new formulations are more expensive and are not commercially available in a number of European countries. In Belgium, the two LT4 branded tablet formulations (L-Thyroxine®, Takeda and Euthyrox®, Merck) are the only TH currently marketed and reimbursed.

Approximately 10% of patients treated with LT4 for hypothyroidism report persisting hypothyroid symptoms despite biochemical euthyroidism [7]. One of the hypothesis to explain this finding is the inability of LT4 to restore normal TH physiology [5], opening the way to the alternative of LT3 treatment. However, current European guidelines, endorsed by Belgian professional societies, recommend monotherapy with LT4 as the first line treatment of hypothyroidism, due to lack of evidence of superiority of LT3 + LT4 treatment with respect to hypothyroid symptoms and quality of life [10]. Nevertheless, persisting symptoms are a strong determinant of the physicians’ prescribing preferences, independent of biochemical control of hypothyroidism [11].

This survey is part of THESIS (Treatment of Hypothyroidism in Europe by Specialists: An International Survey), an international initiative investigating whether differences in practice between 28 European countries impact management of hypothyroidism [12–20]. The aim of our survey was to identify current attitudes on the treatment of hypothyroid and euthyroid patients with thyroid hormones in Belgium.

## Methods

We used an English language web-based survey constructed with Survey Monkey, an open-access platform that provides various question templates. The questionnaire included 26 questions of which 9 related to the professional characteristics of the respondents (Additional File 1). An invitation e-mail, including an electronic link to the questionnaire, was sent to the 160 members of the Belgian Endocrine Society (BES), followed by two reminders between October 15 and December 31, 2020. Anonymized responses were stored electronically. The survey service automatically blocked repeat submissions from the same IP address.

The survey was approved by the Ethics Committee (Comité d’Ethique) Hospitalo-Facultaire des Cliniques Universitaires St-Luc, Université Catholique de Louvain, Brussels, Belgium.

### Statistical analyses

Results are presented as absolute numbers and percentages of respondents. The characteristics of respondents (years of practice < 10 years, 10–30 years, > 30 years, specialty Endocrinology vs. other, and place of practice University center vs. other, shown in Table 1) were compared according to the answers to questions B1 (Indications for TH in biochemically euthyroid patients) and B13 (The use of combined LT4 and LT3 treatment) using a Chi-square test or Fisher’s exact test. Binary logistic regression analysis was performed to identify, among the characteristics of responders (years of practice < 10 years, 10–30 years, > 30 years, specialty Endocrinology vs. other, and place of practice University center vs. other), those that influence the level (high score 5–8 vs low score 1–4) of the score given to each of the eight potential reasons of question B17 (Persisting symptoms in LT4 treated patients achieving normal TSH). All covariates with a *p*-value less than 0.20 in univariate analysis were introduced into a multivariate model. A backward elimination strategy was used to estimate the best model. Analyses were done using SAS V9.4 software (SAS Institute Inc., Cary, NC, USA). All *p*-values are two-sided and values less than 0.05 were considered statistically significant.

**Table 1:** Demographic characteristics of the 80 participants to the survey

Sex, n (%)	
Male	41 (51)
Female	39 (49)
Age, n (%)	
< 40	21 (26)
40–60	45 (56)
> 60	14 (18)
Years in medical practice, n (%)	
< 10	22 (27.5)
10–30	40 (50)
> 30	18 (22.5)
Specialty^a^, n (%)	
Endocrinology	59 (74)
Internal Medicine	14 (17.5)
Pediatric Endocrinology	15 (19)
Nuclear Medicine	8 (10)
Other, including Family Medicine	2 (2.5)
Type of practice^a^, n (%)	
University center	42 (52.5)
Regional hospital	37 (46)
Private clinic	4 (5)
Other, including General practice	7 (9)

^a^ The sum of percentages exceeds 100% because some respondents had > 1 specialty and were employed in more than 1 place

## Results

### Respondent characteristics

All eighty respondents (50% of the BES members), representing five different clinical specialties (Endocrinology, Internal Medicine, Pediatric Endocrinology, Nuclear Medicine and Family Medicine) completed the survey. Endocrinologists represented 74% of the respondents. Four endocrinologists with multiples specialties were characterized as endocrinologists in the statistical analyses.

Forty-six (57.5% of the respondents) were members of other national and/or international endocrine or thyroid associations and nearly half of them (38 participants, 47.5%) practiced in non-university centers. All the participants were clinically active. The demographic characteristics of the respondents are listed in Table 1.

Fifty participants (62.5%) treated hypothyroid patients on a daily basis, 29 (36%) on a weekly basis, whereas only one rarely managed hypothyroid patients. More than 100 hypothyroid patients/year were treated by 32 (40%) respondents, 51–100 annually by 28 (35%), and 20 (25%) treated less than 50 hypothyroid patients/year.

### Choice of thyroid hormones

LT4 was the first treatment of choice for hypothyroidism for all respondents with none opting for LT3, LT4 + LT3 combination or desiccated thyroid extract. Nevertheless, some physicians also use LT3 and LT4 + LT3 combinations in their clinical practice (2 and 7 respondents, respectively). Combination therapy was considered by 20% of respondents for patients with normal serum TSH complaining of persistent symptoms suggestive of hypothyroidism, but this therapy would never be used by 74% of the respondents due to low quality evidence of superiority over LT4 monotherapy. There were no significant associations between use of combination treatment for symptomatic euthyroid patients and years in medical practice or physician working environment. Endocrinologists tended to prescribe LT4+ LT3 treatment more often than other colleagues (26% of endocrinologists vs. 6.3% of the other specialists, *p* = 0.056).

The majority of respondents (92.5%) stated that their patients were using the type of LT4 that they recommended.

### Clinical indications for treatment with thyroid hormones

**Fig. 2:**
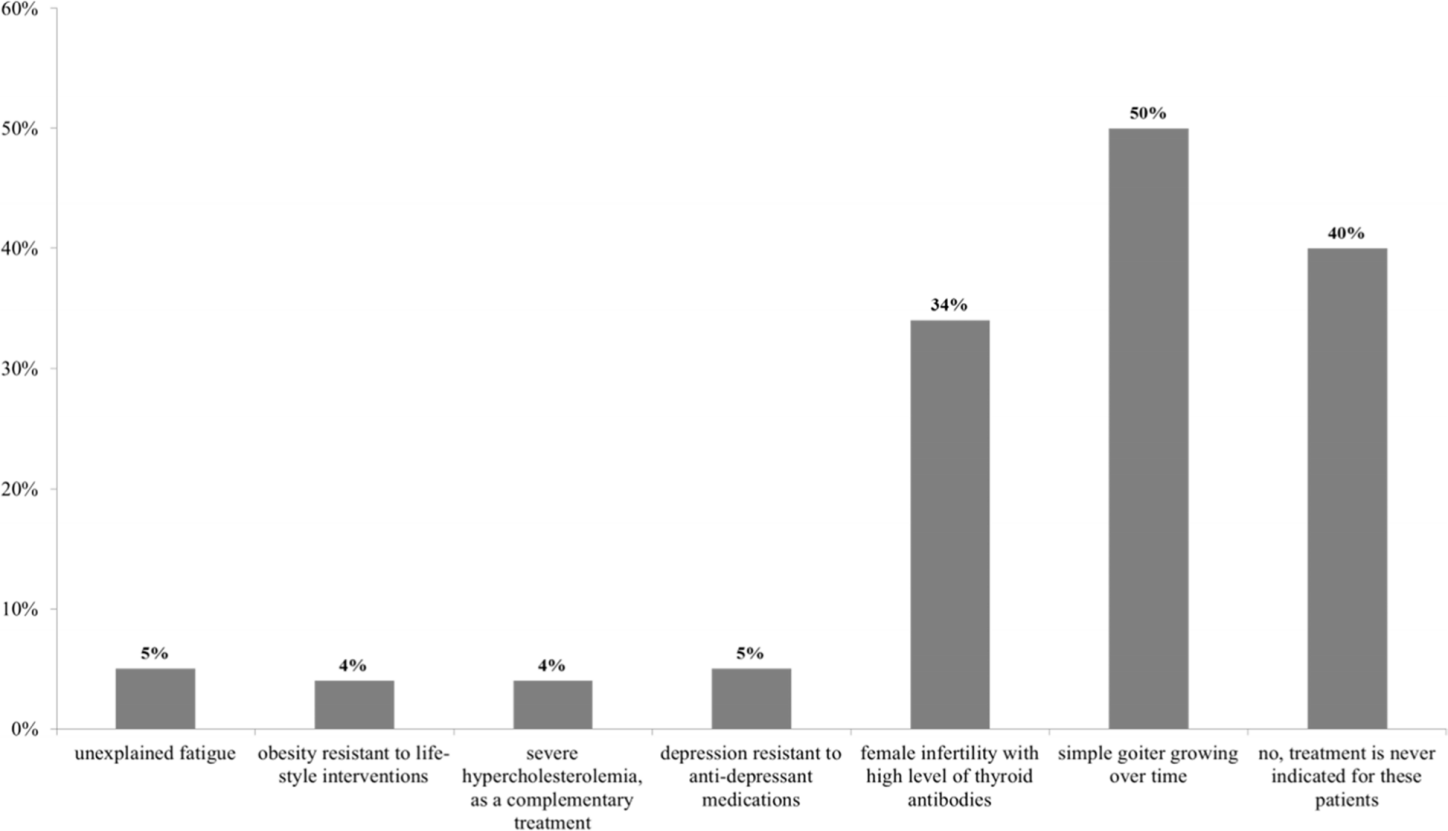
Reasons for prescribing thyroid hormones in euthyroid patients. Multiple answers were possible. Columns represent the percentage of respondents who prescribe LT4 for the given condition

**Fig. 3:**
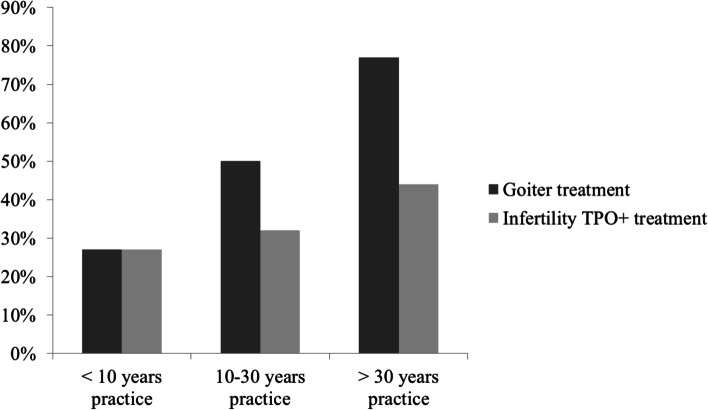
Percentage of respondents, in relation to length of clinical practice, who treat biochemically euthyroid infertile females with high levels of antithyroid antibodies or euthyroid patients with benign goiter growing over time, with thyroid hormones

A significant proportion of participants responded that TH treatment is indicated in biochemically euthyroid but infertile females with high levels of antithyroid antibodies and in euthyroid patients with benign goiter growing over time (34 and 50% of respondents, respectively) (Fig. 2). Other clinical conditions in euthyroid patients (depression resistant to antidepressant medications, obesity resistant to lifestyle intervention, severe hypercholesterolemia and unexplained fatigue) were considered as indications for TH treatment by less than 5%of respondents. Forty percent of respondents never treated euthyroid patients with TH. There were no significant differences, in terms of specialty or type of practice (university vs. other), between physicians who treated or did not treat (for any reason) euthyroid patients. Physicians who had practiced for more than 30 years were more prone to treat than those with shorter clinical practice (41% with less than 10-year practice vs. 60% with 10–30-year practice vs. 83% with more than 30-year practice, *p* = 0.024). This difference was greater for TH treatment of benign euthyroid goiter (27% vs. 50% vs. 77%, respectively, *p* = 0.009) (Fig. 3). Specialty (endocrinology vs. other) and place of practice (university vs. other) did not influence the treatment of benign euthyroid goiter (*p* = 0.638 and *p* = 0.958, respectively).

After initiating LT4 treatment for hypothyroidsm, 57.5% of physicians would re-check serum TSH level after 4 to 6 weeks, and 42.5% after 8 weeks. In case of switching to a different formulation or change from one manufacturer’s LT4 tablet to another, 45% would still re-check the TSH after 4 to 6 weeks, 52.5% after 8 weeks but 2.5% (2 out of 80 individuals) would rely on a clinical evaluation for choice of this interval.

### Use of different LT4 formulations

Several questions explored different clinical scenarios where alternative formulations (soft-gel capsules and liquid solution) could be preferred over classical LT4 tablets in patients taking interfering drugs or food, or presenting with coeliac disease, malabsorption, lactose intolerance, or intolerance to common excipients (Additional File 2). Had alternatives for LT4 tablet formulations been available, only 2 out of 80 (2.5%) participants would consider them for patients achieving biochemical euthyroidism but remaining symptomatic. This proportion was higher for patients with unexplained poor biochemical control of hypothyroidism (13.5%) and gastrointestinal diseases or interfering drugs (10%).

### Use of dietary supplements

Fifty-one percent of respondents would never use dietary supplements. Selenium or iodine were recommended in addition to TH replacement by 7.5% in case of coexisting autoimmune thyroiditis, by 11% for subclinical hypothyroidism and by 30% if requested by the patients.

### Management of symptomatic patients despite achieving a normal TSH

**Fig. 4:**
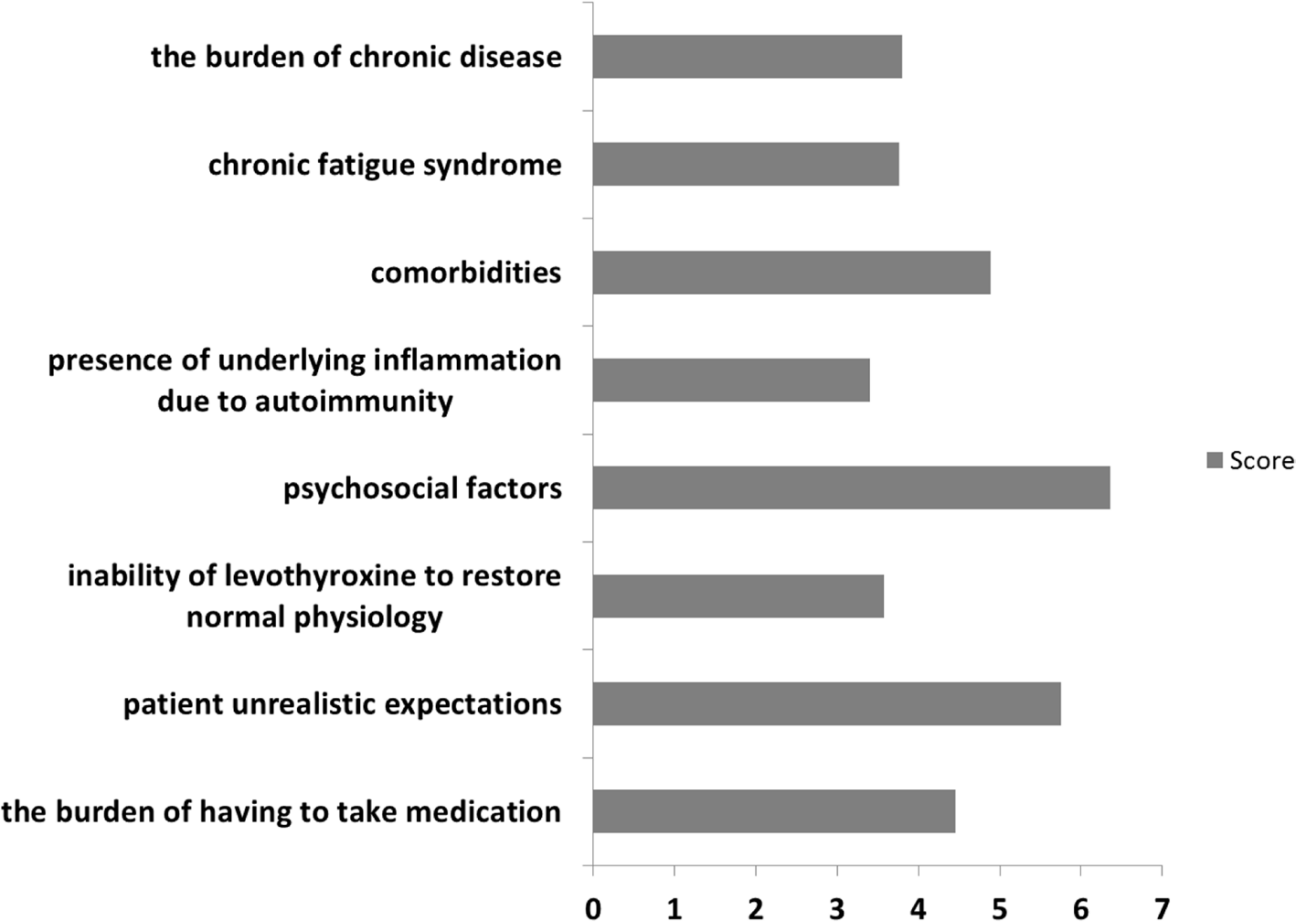
Presumed explanations for persistent symptoms in hypothyroid patients despite achievement of a normal TSH under LT4 treatment. Participants were asked to rank the potential explanations. Higher columns represent greater presumed importance

Most of the respondents considered that the percentage of symptomatic patients despite obtaining a normal TSH is less than 5% (34% of participants) or between 6 and 10% (32.5%). In the opinion of 51% of respondents, this population is unchanged in the last 5 years, while 24% thought it is growing. According to the majority, the most likely explanation for persistent symptoms is psychosocial factors (ranked 6.3 on a scale from 1 to 8), followed by patients’ unrealistic expectations (5.7), presence of comorbidities (4.8), burden of having to take medication (4.4), burden of chronic disease (3.8), chronic fatigue syndrome (3.7), inability of LT4 to restore normal physiology (3.5), and, finally, presence of underlying inflammation due to autoimmunity (3.4) (Fig. 4).

In univariate analyses, the specialty, the type of practice or the years in medical practice did not modify the way the respondents ranked the above reasons, except for patients’ unrealistic expectations which received higher scores from the non-endocrinologists (odds ratio 3.27; 95% CI: 1.10–9.68; *p* = 0.032). This difference was maintained in a multivariate analysis.

## Discussion

This is the first survey on the use of TH by Belgian specialists. As elsewhere in Europe [12–20] and in agreement with international guidelines, the survey confirmed that LT4 is the treatment of choice for Belgian hypothyroid patients. However, a significant proportion of respondents also use TH for more controversial indications, such as infertile euthyroid TPOAb positive women and a growing benign goiter. A similar or even greater preference for the treatment of infertile euthyroid women in the presence of thyroid autoimmunity was reported by French (31.7%), Greek (31.9%), Romanian (36.4%), Italian (37.3%), Danish (42.1%), Swedish (47.3%), Spanish (48.5%) and Polish (63.4%) endocrinologists who responded to this question, respectively [12–20], although the limited available evidence does not support this approach. In the case of assisted reproductive technology, all large randomized controlled trials in this population concluded that there is no benefit from LT4 treatment [21, 22], which was confirmed in the recent TABLET trial including euthyroid TPOAb positive women with a medical history of (recurrent) miscarriage or receiving treatment for infertility [23]. The ETA guidelines on thyroid disorders prior to and during assisted reproduction published in 2021 [24] state that association with adverse fertility outcomes appears to emerge at TSH levels above 4.0 mIU/L, when LT4 treatment should be started. Nevertheless, limited data indicate that LT4 treatment optimized the ovarian reserve, fertilization and embryo development. Therefore, it has been suggested to consider LT4 treatment in infertile women with thyroid autoimmunity and serum TSH > 2.5 mIU/L on a case-by-case basis, taking into account factors such as ovarian causes of subfertility, older age (> 35 years), a history of recurrent miscarriage, or high levels of thyroid antibodies [24].

In contrast to current evidence, a surprisingly high proportion of Belgian specialists (50%) consider TH treatment in patients with a euthyroid benign goiter growing over time, a rate similar to Bulgarian (55%), Polish (40.9%) and French (40.2%) colleagues [14, 15, 19], but higher than 24.2% of Greek [18], 21.2% of Spanish [17],18% of Italians [12],14% of Swedish [20] and 12.5% of Danish endocrinologists who responded to this question [13]. This response may be related to adherence of older endocrinologists to outdated clinical practices, no longer recommended by recent guidelines [25]. Among these outdated ideas, one may be reminded of the misbelief that LT4 is a source of iodine in previously iodine-deficient areas. Euthyroid goiter is more prevalent in populations with low or borderline iodine intake and evolves from a diffuse thyroid enlargement at younger ages to an autonomous nodular disease in elderly. A number of studies show that goiter size is at best marginally reduced by LT4 [26, 27], and that lowering TSH is associated with osteoporosis and cardiovascular morbidity [28] and mortality [29] in a dose-dependent way [30]. A diffuse goiter might benefit from TSH-lowering treatment [27], but surgery or radioiodine are more effective [26, 27, 31]. In Belgium, radioiodine is not traditionally used for the treatment of nontoxic goiter. Although radioiodine does lead to significant thyroid volume reduction, relatively high activities of radioiodine are needed [32], because of a frequent finding of a low thyroid radioiodine uptake, especially since the iodine salt addition policies have been largely implemented. Moreover, in Belgium, radioiodine can be given in outpatient clinic only up to 15 mCi. Thyroid radioiodine uptake can be enhanced by using recombinant TSH [27] but the product is not reimbursed in Belgium for this indication. Belgian specialists might also fear that thyroid autoimmunity, including thyroid-associated orbitopathy, might be induced or exacerbated by radioiodine administration. These limitations of alternative treatments may explain the choice of Belgian specialists for thyroid hormones in the management of simple euthyroid goiter. At contrast, in Denmark, where radioiodine is the preferred treatment for nontoxic goiter [33], only one in ten specialists recommend thyroid hormone for this indication [13].

Contrary to other European countries, LT3 and combination of LT4 + LT3 are not available on the Belgian market, but may be ordered from other European countries. This might be the reason why less than 10% of Belgian specialists use these therapies, compared with e.g. 58.6% of their Danish colleagues [13]. However, a higher number of respondents (20% of Belgians) are willing to switch to combination treatment in patients with normal TSH and persisting symptoms of hypothyroidism on LT4, a tendency noted in other THESIS surveys (25% of Greeks [18], 32.2% of Polish [15], 40% of Italians [12]. This proportion was even higher in Danish (71%) and Swedish (78.5%) specialists [13, 20], despite the lack of evidence of superiority of combined treatment over LT4 and lack of long-term safety data. The preference towards combined treatment was more frequent in Belgian endocrinologists than other Belgian physicians, but was not influenced by the physician working environment or years of practice. We can only speculate that the endocrinologists may be more aware of the LT4 monotherapy controversies and the alternative of combination LT4 + LT3 treatment. As an example, in Polish THESIS, physicians who believed that persistent symptoms are not due to inability of LT4 to restore normal physiology were less likely to prescribe combination LT4 + LT3, than physicians who believed that LT4 does not restore normal physiology [14]. A recent expert consensus stated that a new clinical trial of LT4 + LT3 therapy is justified but should be correctly designed to address unsettled questions, such as: effect of deiodinase and TH transporter polymorphisms, inclusion of patients taking significant doses of LT4 and use of multiple daily doses or slow-release LT3 [34]. In the meantime, specialists should be aware that current guidelines suggest that a trial of LT4 and LT3 combination treatment may be considered only in symptomatic patients achieving biochemical euthyroidism for at least six months and after the exclusion of interfering conditions and comorbidities [10], the latter being very prevalent in patients with hypothyroidism [35]. Furthermore, combination treatment should be discontinued after a few months if there is no clinical improvement.

Although hypothyroid patients dissatisfied with their treatment represent a minority, our survey suggests, as other investigations of this type [12–14, 20], that their proportion is either stable or increasing over time. The reasons for this dissatisfaction are much debated and include a number of contributors other than hypothyroidism per se, among them psychosocial factors, comorbidities and aspects of the patient-physician communication [5, 36, 37]. In this respect, our survey did not enquire about patient compliance with treatment.

The clinical experience with LT4 formulations other than tablets is very limited in Belgium. Nevertheless, the percentage of specialists expecting no major clinical changes for a symptomatic euthyroid patient switching to another formulation was similar to the one observed in Italy (42.5% Belgians vs. 50.3% Italians). Italian specialists are familiar with soft-gel capsules and liquid solution alternatives which they prefer in patients with specific conditions (interfering drugs, actual or suspected malabsorption, inability to take LT4 in the fasting state, unexplained poor biochemical control of hypothyroidism) [12]. In these conditions, the use of alternative LT4 formulations may also be favored by some Belgian specialists, but more than one in three were unable to formulate an opinion in the absence of clinical experience.

Selenium supplementation in addition to thyroid hormones was recommended by some of our survey respondents, although there is insufficient evidence to support this approach. A decline in thyroid autoantibodies in patients treated with selenium [38], does not offer documented clinical efficacy in chronic autoimmune thyroiditis [39]. Nevertheless, one third of the respondents prescribed dietary supplements, including selenium, at the patients’ request. This trend was already noticed among physicians in Europe [40], and is not recommended by current guidelines on management of hypothyroidism [41].

The strength of our survey is an acceptable response rate of 50%, comparable to that of a previous national Belgian survey on thyroid nodule management [42], and one of the highest of all THESIS surveys (vs. 25.5% in France, 25.8% in Spain, 28.2% in Sweden, 31.2% in Denmark, 39.3% In Italy, 42.4% in Romania) [12, 13, 16, 17, 19, 20], despite the constraints of the ongoing Covid-19 pandemic. Physician surveys performed anonymously can reveal important habits and practices that are not evidence-based, potentially harmful, and identify the extent to which these may need to be prioritized as educational unmet needs that require to be addressed by professional organizations and societies. Although the absolute number of participants in our survey is limited, they were experienced with TH treatment and mostly of them treated at least as many hypothyroid patients on a daily basis compared to larger countries participating to THESIS initiative (62.5% of Belgians vs. 57.8% of Italians, 49.1% of Swedish or 34.2% of Danish) [12, 13, 20]. Moreover, our survey, as the majority of THESIS surveys, identified similar trends and deviations in the treatment with TH, as the treatment of euthyroid TPO+ infertile women and the preference for combined LT4 + LT3 treatment in hypothyroid patients with persistent symptoms. In this respect, data generated by our survey could be relevant to many other countries with similar populations and health care systems.

Our survey has also limitations. It can be argued that physician surveys record opinions rather than real practice. While this is true, physician surveys are not worthless. Indeed in the field of hypothyroidism physician surveys have been published and cited widely, and have helped expand our knowledge about management of hypothyroidism [11, 43–46]. The survey targeted members of the BES, mostly endocrinologists, although several medical specialties are involved in TH treatment in Belgium. According to the National Institute of Diseases (INAMI-RIZIV, https://www.inami.fgov.be), 88% of thyroid hormones prescriptions made in Belgium in 2019 were made by general practitioners (GPs). However, this is not an isolated situation. For example, most Swedish patients with hypothyroidism are treated by GPs [20] and hypothyroidism is managed both in primary and secondary care in Denmark [12]. Although GPs were under-represented in THESIS surveys, it is likely that they follow recommendations issued by specialists via national guidelines or Continuing Medical Education sessions. As in Sweden, Belgian specialists and GPs are working in close relationship for the treatment of patients with thyroid hormones. Therefore, the indications for the prescription of TH by specialists probably impact and influence those of GPs and are worth studying.

As the proportion of specialists working in a university center was over-represented, we cannot exclude, as another limitation to our study, that the respondents represented physicians treating a high proportion of dissatisfied patients in search of alternatives to standard TH treatment.

Although our survey reflects mainly the experience with levothyroxine tablet monotherapy, that is the treatment that the vast majority of hypothyroid patients receive, and the evidence base for clinical outcomes is far more robust than other thyroid hormones.

In conclusion, in Belgium TH are generally used according to current guidelines, but we did note, as most of THESIS surveys, deviations from evidence-based recommendations. Thyroid autoimmunity in euthyroid infertile women and euthyroid benign goiter were common indications for LT4 treatment, constituting practices in need of further scrutiny as unjustified thyroid hormones treatment can lead to iatrogenic hyperthyroidism and be dangerous. In Belgium, choice of TH is relatively uninfluenced by demographic respondent variables such as clinical specialty, type of practice or years of medical practice, and reflects the availability of drug formulations and type.

## Supplementary Information

**Additional File 1.** Belgium Hypothyroid Survey Questionnaire.

**Additional File 2.** Preference for different LT4 formulations in different clinical situations.

## Data Availability

The datasets used and/or analysed during the current study are available from the corresponding author on reasonable request.
