# Supplementary material for: Use of thyroid hormones in hypothyroid and euthyroid patients: a THESIS* survey of Belgian specialists *THESIS: treatment of hypothyroidism in Europe by specialists: an international survey
Source: Thyroid Res. 2022 Mar 5;15:3. doi: 10.1186/s13044-022-00121-9 (PMC8897091; doi:10.1186/s13044-022-00121-9)
Supplement: Supplementary file 2 — Additional File 2. Preference for different LT4 formulations in different clinical situations. [file 13044_2022_121_MOESM2_ESM.docx]

|  | Tablets  N (%) | Soft-gel capsules  N (%) | Liquid solution  N (%) | I expect no major changes with different formulations  N (%) | I don't know, as not all of the above formulations are available  N (%) |
| --- | --- | --- | --- | --- | --- |
| B5. Interfering drugs may influence the stability of therapy. Which LT4 preparation is in your experience least likely to be subject to variable absorption? | 22 (27.5) | 4 (5) | 7 (9) | 6 (7.5) | 41 (51) |
| B6. Which of the following preparations of LT4 would you prescribe in case of first diagnosis of hypothyroidism when the patient self-reports intolerance to various foods raising the possibility of celiac disease, malabsorption, lactose intolerance, or intolerance to common excipients | 41 (51) | 5 (6) | 3 (4) | 4 (5) | 27 (34) |
| B7. Which of the following preparations of LT4 would you prescribe for a patient established on LT4 who has unexplained poor biochemical control of hypothyroidism? | 18*(22.5) | 6 (7.5) | 5 (6) | 17 (21) | 34 (43) |
| B8. Which of the following preparations of LT4 would you prescribe for a patient with poor biochemical control who is unable (due to busy lifestyle) to take LT4 fasted and separate from food/drink? | 28 (35) | 5 (6) | 6 (7.5) | 11 (14) | 30 (37.5) |
| B9. Which of the following preparations of LT4 would you prescribe for a patient established on LT4 tablets who has good biochemical control of hypothyroidism but continues to have symptoms? | 18*(22.5) | 1 (1) | 1 (1.5) | 34 (42.5) | 26 (32.5) |

*Tablets from another manufacturer
